# Transient receptor potential ankyrin 1 that is induced in dorsal root ganglion neurons contributes to acute cold hypersensitivity after oxaliplatin administration

**DOI:** 10.1186/s12990-015-0072-8

**Published:** 2015-11-13

**Authors:** Ken Yamamoto, Noriko Chiba, Terumasa Chiba, Toshie Kambe, Kenji Abe, Kazuyoshi Kawakami, Iku Utsunomiya, Kyoji Taguchi

**Affiliations:** Department of Medicinal Pharmacology, Showa Pharmaceutical University, 3-3165 Higashitamagawagakuen, Machida, Tokyo 194-8543 Japan; Department of Pharmacology, Showa Pharmaceutical University, 3-3165, Machida, Tokyo 194-8543 Japan; Department of Pharmacology, School of Pharmaceutical Sciences, Ohu University, 31-1 Tomitamachi, Koriyama, Fukushima 963-8611 Japan; Department of Pharmacy, Cancer Institute Hospital, 3-10-6 Ariake, Koto-ku, Tokyo, 135-8550 Japan; Department of Pharmacotheraputics, Showa Pharmaceutical University, 3-3165, Machida, Tokyo 194-8543 Japan

**Keywords:** Oxaliplatin, Transient receptor potential ankyrin 1, p38 mitogen-activated protein kinase, Acute cold hyperalgesia, Peripheral neuropathic pain

## Abstract

**Background:**

Peripheral cold neuropathic pain is a serious side effect of oxaliplatin treatment. However, the mechanism of oxaliplatin-induced cold hyperalgesia is unknown. In the present study, we investigated the effects of oxaliplatin on transient receptor potential ankyrin 1 (TRPA1) in dorsal root ganglion (DRG) neurons of rats.

**Results:**

Behavioral assessment using the acetone spray test showed that 3 and 6 mg/kg oxaliplatin (i.p.) induced acute cold hypersensitivity after 1, 2, 4, and 7 days. Real-time PCR showed that oxaliplatin (6 mg/kg) significantly increased TRPA1 mRNA expression in DRGs at days 1, 2, and 4. Western blotting revealed that oxaliplatin significantly increased TRPA1 protein expression in DRGs at days 2, 4, and 7. Moreover, in situ hybridization histochemistry revealed that most TRPA1 mRNA-labeled neurons in the DRGs were small in size. Oxaliplatin significantly increased co-localization of TRPA1 expression and isolectin B4 binding in DRG neurons. Oxaliplatin induced a significant increase in the percent of TRPA1 mRNA-positive small neurons in DRGs at days 1, 2, and 4. In addition, we found that intrathecal administration of TRPA1 antisense, but not TRPA1 mismatched oligodeoxynucleotides, knocked down TRPA1 expression and decreased oxaliplatin-induced cold hyperalgesia. Double labeling showed that p-p38 mitogen-activated protein kinase (MAPK) was co-expressed in TRPA1 mRNA-labeled neurons at day 2 after oxaliplatin administration. Intrathecal administration of the p38 MAPK inhibitor, SB203580, significantly decreased oxaliplatin-induced acute cold hypersensitivity.

**Conclusions:**

Together, these results demonstrate that TRPA1 expression via activation of p38 MAPK in DRG neurons, at least in part, contributes to the development of oxaliplatin-induced acute cold hyperalgesia.

## Background

Oxaliplatin is a platinum-based chemotherapeutic agent that is effective against advanced colorectal cancer. However, this drug induces painful peripheral neuropathy as a dose-limiting side effect. Oxaliplatin-induced neurotoxicity manifests as rapid-onset neuropathic symptoms that are exacerbated by cold exposure and as chronic neuropathy that develops after several treatment cycles [1, 2]. In rodents, a single injection of oxaliplatin induces cold and mechanical allodynia [3–5]. Oxaliplatin is metabolized to oxalate and dichloro (1,2-diaminocyclohexane)platinum [Pt(dach)Cl_2_] [6]; oxalate is related to cold hyperalgesia, and Pt(dach)Cl_2_ induces the mechanical allodynia [2].

Temperature is sensed by a subpopulation of peripheral primary afferent fibers known as thermoreceptors. Several candidate thermo-sensor molecules have been identified that belong to the transient receptor potential (TRP) ion channel family [7]. Two of these ion channels, termed TRP ankyrin 1 (TRPA1) and TRP melastatin 8 (TRPM8), have been proposed to function as cold transducers [8–10] and have been identified as cold-sensitive ion channels. TRPM8 is activated by menthol and cooling, with an activation temperature of approximately 25–28 °C [11, 12]. TRPA1, which is expressed by sensory neurons, is activated at approximately 17 °C, a temperature that is reported as painfully cold by humans [13–15]. Recently, Nassini et al. reported that a single dose of oxaliplatin produces mechanical and cold hyperalgesia in rats, and this effect is selectively attenuated by a TRPA1 antagonist [16]. In addition, mechanical and cold hyperalgesia are absent in TRPA1-deficient mice [17]. Thus, TRPA1 may contribute to acute cold hypersensitivity evoked by administration of oxaliplatin.

Activation of p38 mitogen-activated protein kinase (MAPK) contributes to the development and maintenance of inflammatory and neuropathic pain [18–20]. p38 MAPK signaling can be activated in the DRG by administration of capsaicin or noxious thermal stimuli [21]. Activated p38 MAPK in the DRG is thought to play an important role in oxaliplatin-induced acute cold hypersensitivity. However, the mechanisms of oxaliplatin-induced acute cold hypersensitivity and activation of the p38 MAPK pathway have not yet been evaluated, and the mechanisms underlying the up-regulation of oxaliplatin-induced TRPA1 are still poorly understood. Thus, because TRPA1 is a sensor of cold temperatures, we hypothesized that up-regulation of TRPA1 via a p38 MAPK pathway mediates cold hyperalgesia induced by oxaliplatin. The aim of this study was to investigate the involvement of TRPA1 and p38 MAPK in DRGs in oxaliplatin-induced acute cold hypersensitivity.

## Results

### Effects of oxaliplatin on acute cold hypersensitivity

**Fig. 1:**
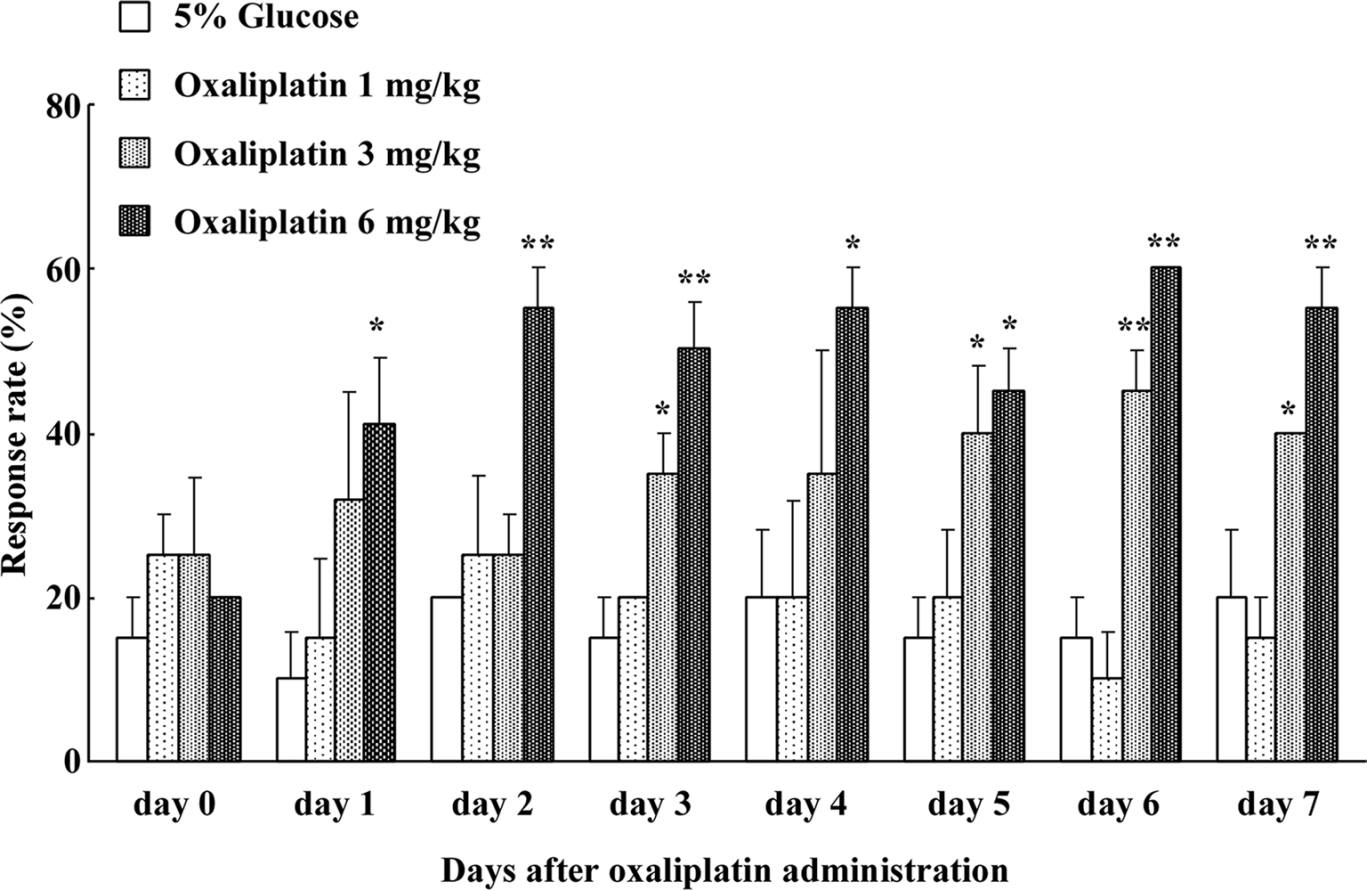
Time course of acute cold hypersensitivity after oxaliplatin administration. The acetone spray test was used to measure cold response (1, 3, and 6 mg/kg, i.p.) in rats at days 1–7 after oxaliplatin administration. Data are the mean ± SEM of n = 8–10 rats. *P < 0.05, **P < 0.01, two-way ANOVA with Dunnett’s post hoc analysis compared to control (5 % glucose)

We first investigated the effect of oxaliplatin (1, 3, and 6 mg/kg, i.p.) on acute cold hypersensitivity. Before the first single dose of oxaliplatin, we found no significant differences in the number of withdrawal responses in any groups in the acetone spray test. Oxaliplatin (1 mg/kg, i.p.) did not affect the paw withdrawal response rate in the acetone spray test. However, the response rate was significantly increased with 3 and 6 mg/kg oxaliplatin in the acetone spray test at days 1–7 after a single dose of oxaliplatin compared to 5 % glucose treatment (Fig. 1).

### Effect of oxaliplatin on expression of TRPA1 mRNA in DRG neurons

**Fig. 2:**
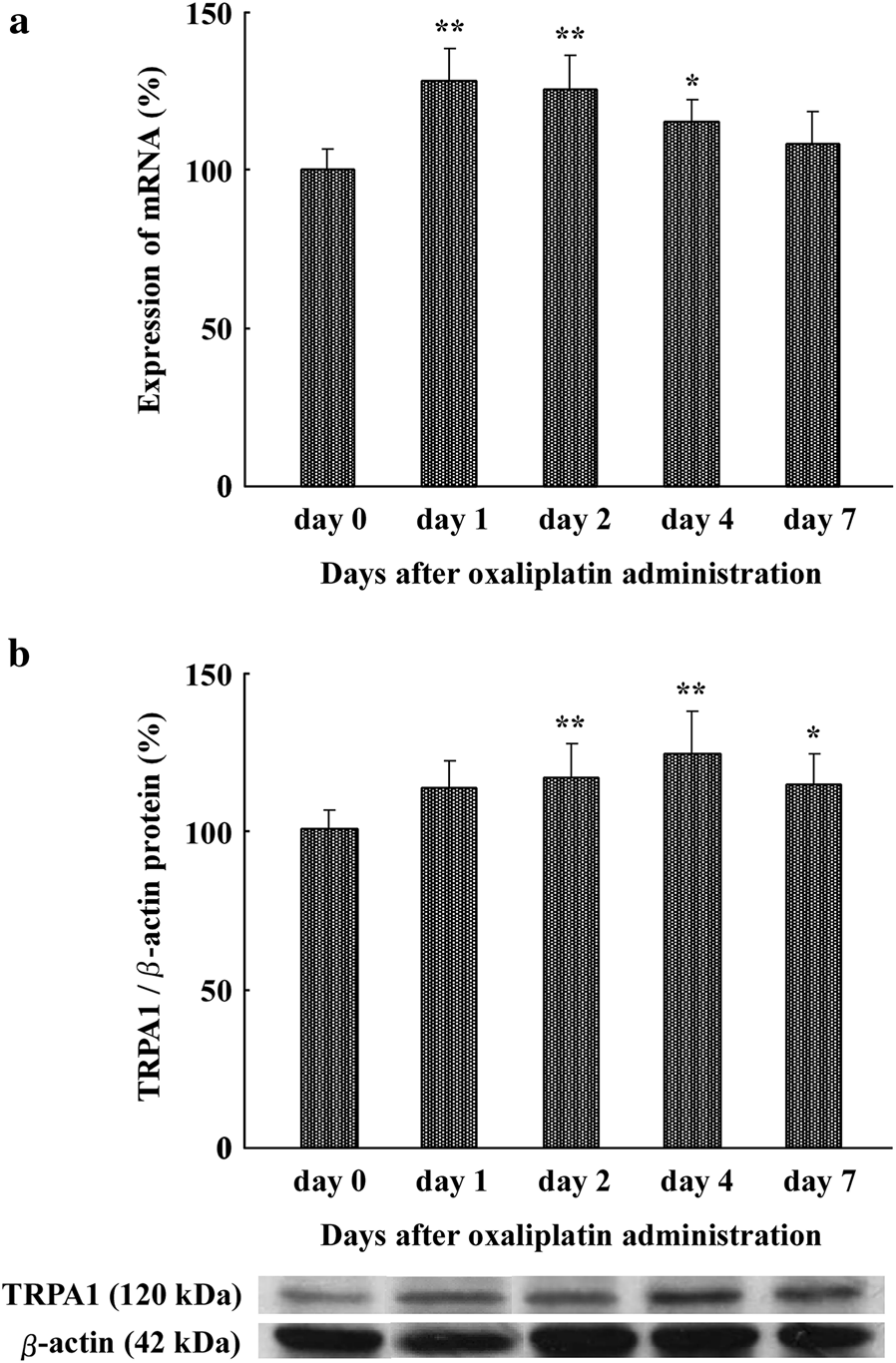
Oxaliplatin administration increases TRPA1 mRNA and protein in DRGs. **a** Effect of oxaliplatin (6 mg/kg, i.p.) on TRPA1 mRNA expression in rat DRGs (L_4–6_) at days 0 (before administration), 1, 2, 4, and 7 was measured. TRPA1 expression was normalized to β-actin expression. Histograms show the relative amount of TRPA1 mRNA in oxaliplatin-treated rats compared with day 0. Data are the mean ± SEM. n = 4 for oxaliplatin administration. *P < 0.05, **P < 0.01 versus day 0. **b** Effect of oxaliplatin (6 mg/kg, i.p.) on TRPA1 protein expression in rat DRGs (L_4–6_). TRPA1 and β-actin protein in DRGs at days 0 (before administration), 1, 2, 4, and 7 was measured. TRPA1 expression was normalized to β-actin expression. Histograms show the relative amount of TRPA1 protein in oxaliplatin-treated rats. The western blot shows representative data. Data are the mean ± SEM. n = 4 each for 5 % glucose and oxaliplatin administration. *P < 0.05, **P < 0.01 versus day 0

We investigated the expression of TRPA1 mRNA during acute cold hypersensitivity after oxaliplatin administration. We removed DRGs (L_4–6_) at days 1, 2, 4, and 7 after the start of 6 mg/kg oxaliplatin, and TRPA1 mRNA expression was quantified with RT-PCR. As shown in Fig. 2a, 6 mg/kg oxaliplatin significantly increased TRPA1 mRNA expression in DRGs at days 1 (127.9 ± 10.4 %, n = 4, P < 0.01), 2 (125.1 ± 10.8 %, n = 4, P < 0.01), and 4 (114.9 ± 7.3 %, n = 4, P < 0.05) compared to day 0 (100.0 ± 6.3 %). TRPA1 mRNA expression was maximally increased at day 1 and returned to control levels 7 days after administration of oxaliplatin (Fig. 2a).

### Effect of oxaliplatin on TRPA1 protein expression

We removed DRGs (L_4–6_) at days 1, 2, 4, and 7 after the start of 6 mg/kg oxaliplatin administration, and TRPA1 protein expression was quantified with western blotting. As shown in Fig. 2b, 6 mg/kg oxaliplatin significantly increased TRPA1 protein expression in DRGs at days 2 and 4. Protein expression at day 7 had begun to decrease, but remained significantly higher than at day 0. Up-regulation of TRPA1 protein was significant at day 2 (116.1 ± 10.7 %, n = 4, P < 0.01) and maximal at day 4 (123.9 ± 13.3 %, n = 4, P < 0.01; Fig. 2b) compared to day 0 (100.0 ± 5.8 %).

### Oxaliplatin increases TRPA1 mRNA-labeled neurons as seen with in situ hybridization histochemistry (ISHH)

**Fig. 3:**
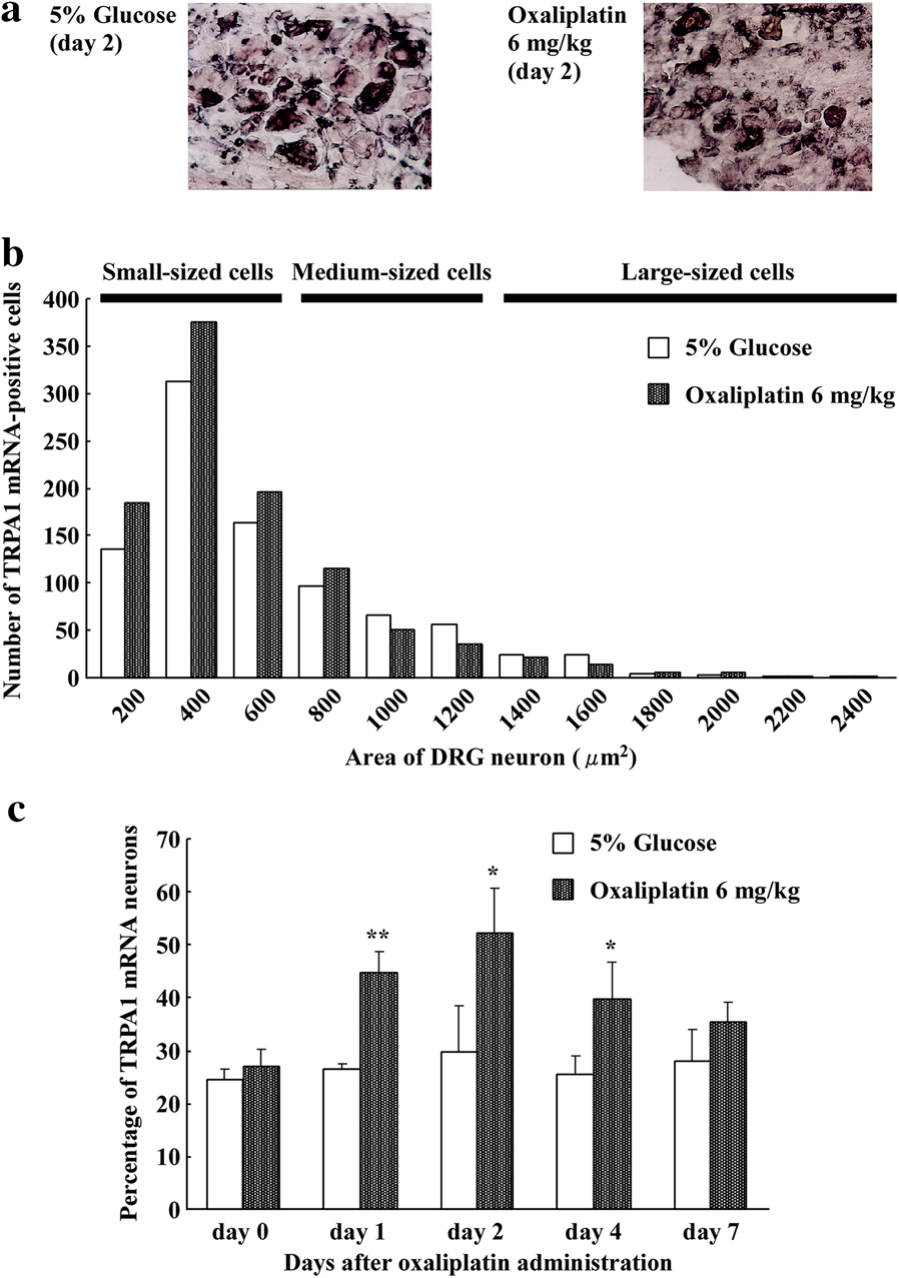
Oxaliplatin increases TRPA1 mRNA as seen with in situ hybridization histochemistry in the DRG. Effect of oxaliplatin (6 mg/kg, i.p.) on TRPA1 mRNA expression in rat DRG (L_4–6_) neurons. **a** Photomicrographs showing in situ hybridization for TRPA1 mRNA at day 2 after oxaliplatin (6 mg/kg, i.p.). TRPA1 mRNA-expressing small-sized neurons were more frequently observed after oxaliplatin treatment. **b** Size distribution of TRPA1-expressing neurons in DRGs in 5 % glucose- and oxaliplatin-treated rats. TRPA1 expression was distributed in all sizes of neurons, but most TRPA1 expression was found in small DRG neurons. **c** Histogram shows the percent of TRPA1-positive neurons relative to the total neurons. TRPA1 mRNA-expressing neurons were more frequently observed at days 1, 2, 4, and 7 after oxaliplatin administration (6 mg/kg, i.p.) compared to 5 % glucose-treated animals. Data are the mean ± SEM. n = 4 each for 5 % glucose and oxaliplatin administration. *P < 0.05, **P < 0.01 versus 5 % glucose

ISHH revealed that most TRPA1 mRNA-labeled neurons in the DRGs (L_4–6_) were small or medium in size (Fig. 3a), consistent with previous studies [9–11]. Most TRPA1 mRNA-positive DRG neurons in oxaliplatin-treated rats were small-sized neurons compared to 5 % glucose treatment (Fig. 3b). Thus, oxaliplatin increased the number of small-diameter DRG neurons that express TRPA1 mRNA. Using computerized image analysis, we found that oxaliplatin (6 mg/kg) induced a significant increase in the percentage of TRPA1 mRNA-positive DRG neurons at days 1 (45.5 ± 3.9 %, n = 4, P < 0.01), 2 (53.0 ± 8.7 %, n = 4, P < 0.05), and 4 (40.4 ± 7.0 %, n = 4, P < 0.05). The percent of TRPA1 mRNA-positive neurons gradually declined, returning to control levels by day 7 (Fig. 3c). These changes in TRPA1 were also confirmed with RT-PCR (Fig. 2). The results indicated that oxaliplatin increased the percent of TRPA1 mRNA-positive neurons as a result of up-regulation of TRPA1 expression in small-diameter DRG neurons in particular.

### TRPA1 gene knockdown prevents oxaliplatin-induced acute cold hypersensitivity

**Fig. 4:**
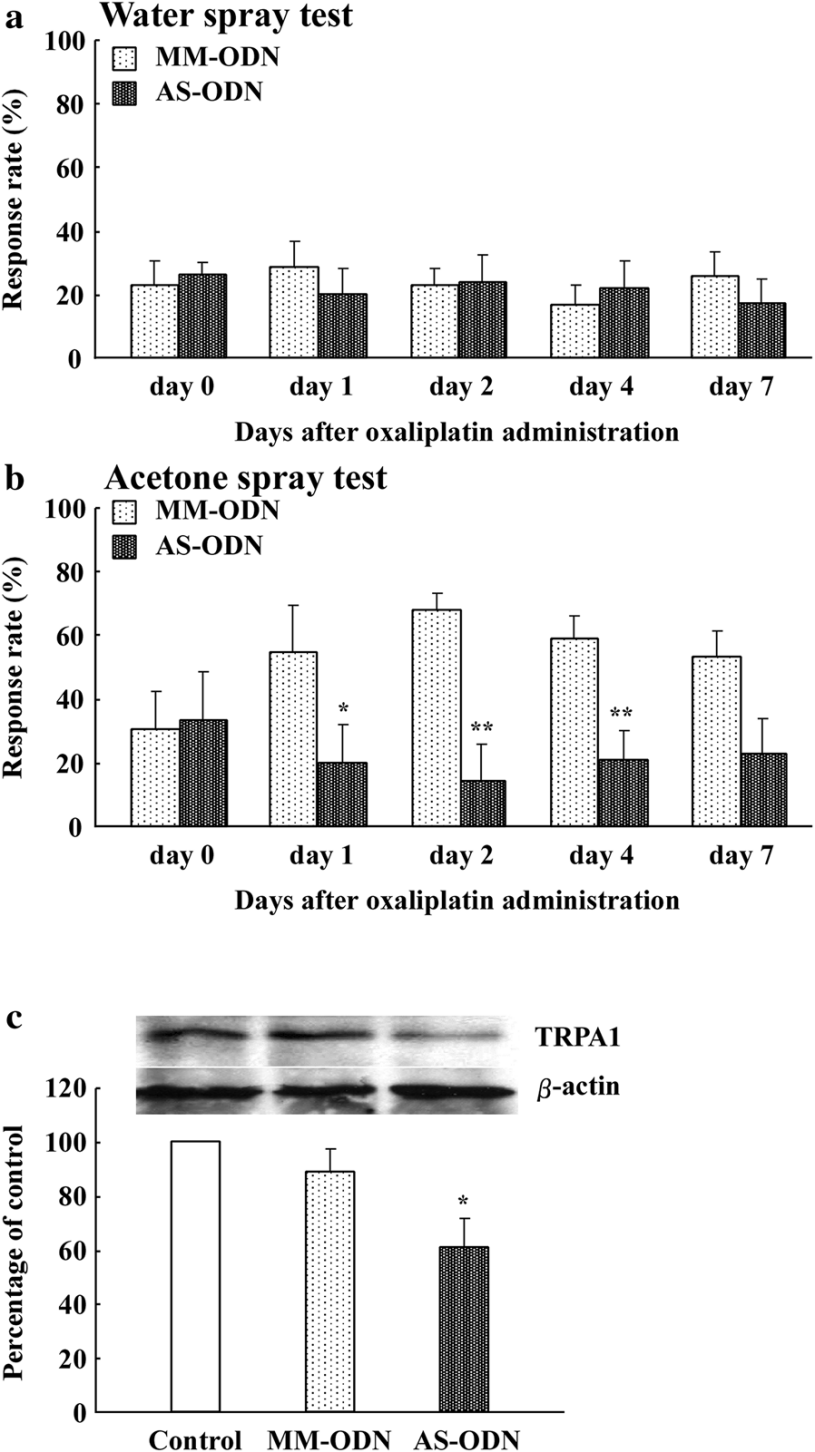
Effect of oxaliplatin on cold hypersensitivity after TRPA1 knockdown in the acetone spray test and water spray test. Intrathecal administration of TRPA1 AS-ODN prevents cold hypersensitivity induced by oxaliplatin (6 mg/kg, i.p.) as seen with the acetone spray test, but not the water spray test. The water spray test (22 °C) and acetone spray test at days 1, 2, 4, and 7 after oxaliplatin were measured in TRPA1 AS-ODN and TRPA1 MM-ODN treatment groups. **a** Histograms show the mean ± SEM of cold hypersensitivity at days 0 (before administration), 1, 2, 4, and 7 after oxaliplatin administration in the water spray test (22 °C). **b** Histograms show cold hypersensitivity at days 0 (before administration), 1, 2, 4, and 7 after oxaliplatin administration in the acetone spray test. Data are the mean ± SEM. n = 4 for oxaliplatin administration. *P < 0.05, **P < 0.01 versus TRPA1 MM-ODN treatment groups. **c** The TRPA1 AS-ODN was effective at reducing TRPA1 protein expression. TRPA1 and β-actin protein in DRGs at day 2 after oxaliplatin administration (6 mg/kg, i.p.) was measured. TRPA1 expression was normalized to β-actin expression. Histograms show the relative amount of TRPA1 protein in oxaliplatin (6 mg/kg, i.p.)-treated rats. The western blot shows representative data. Data are the mean ± SEM. n = 4 for oxaliplatin administration. *P < 0.05 versus TRPA1 MM-ODN treatment groups

Our results suggest that acute cold hypersensitivity after oxaliplatin administration is critically dependent on functional TRPA1 in DRG neurons. We therefore predicted that selective knockdown of TRPA1 expression would prevent oxaliplatin-induced acute cold hypersensitivity. To test this, rats were intrathecally treated with either antisense oligodeoxynucleotides (AS-ODN) targeting TRPA1 or control mismatched oligodeoxynucleotides (MM-ODN) for 3 days before oxaliplatin (6 mg/kg) administration. The paw withdrawal response rate in the TRPA1 AS-ODN and MM-ODN groups was not different in the water spray test (22 °C) (Fig. 4a). The increase in oxaliplatin-induced cold hypersensitivity in the paw withdrawal response rate in the acetone spray test was significantly less in the TRPA1 AS-ODN group at days 1, 2, and 4 than in the MM-ODN group (Fig. 4b). As shown in Fig. 4b, the TRPA1 AS-ODN group showed a significant decrease in the paw withdrawal response rate at days 1 (54.3 ± 14.4 vs. 20.0 ± 11.4 %, n = 4, P < 0.05), 2 (67.3 ± 5.4 vs. 14.3 ± 10.4 %, n = 4, P < 0.01), and 4 (58.4 ± 7.4 vs. 20.9 ± 9.0 %, P < 0.01, n = 4). TRPA1 MM-ODN did not affect oxaliplatin-induced acute cold hypersensitivity in rats. We also confirmed that the level of TRPA1 protein in the DRGs of the TRPA1 AS-ODN-treated rats was significantly lower than in the MM-ODN-treated rats (Fig. 4c).

### The effect of oxaliplatin on TRPA1 co-localization with isolectin B4 binding in DRG neurons

**Fig. 5:**
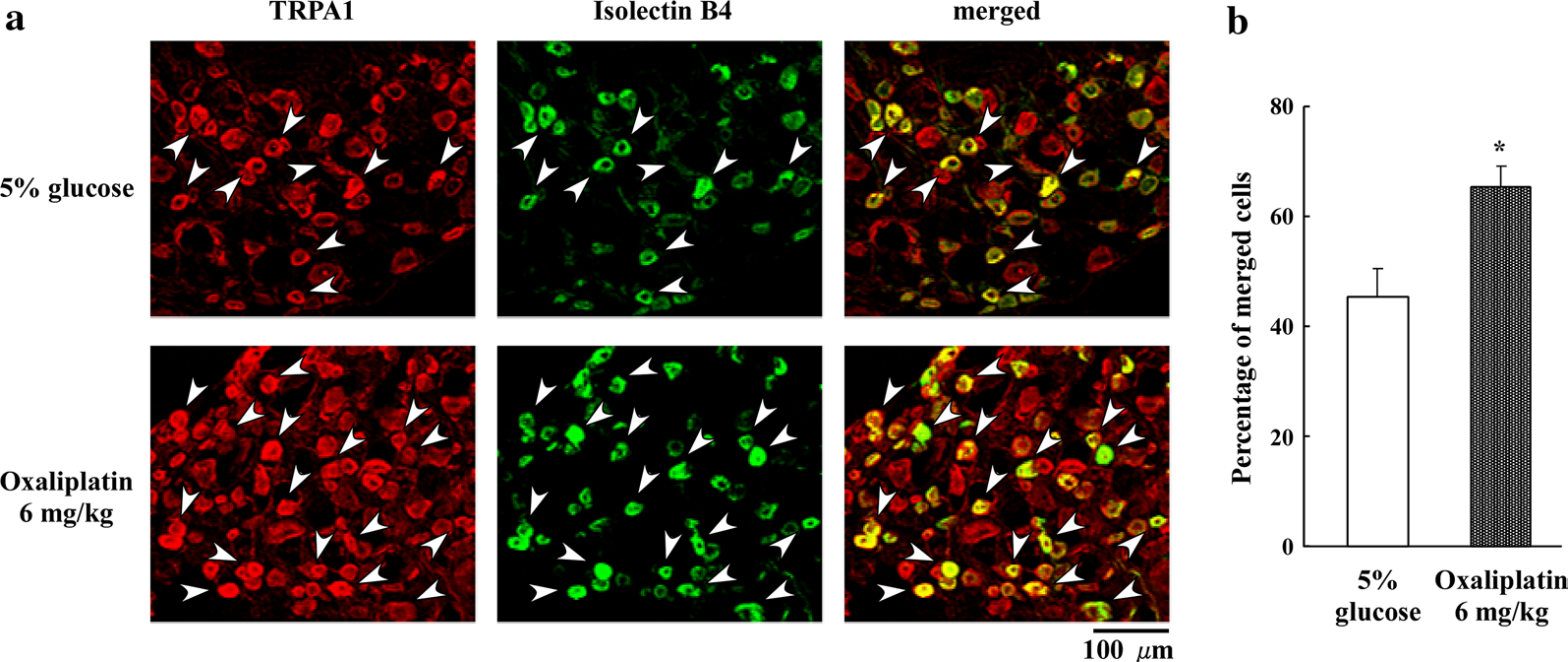
Oxaliplatin increases TRPA1 protein expression as seen with immunohistochemistry in the DRG. **a** Effect of oxaliplatin (6 mg/kg, i.p.) on co-localization of TRPA1 protein expression with isolectin B4 binding in rat DRG (L_4–6_) neurons. The photomicrographs show representative data. Co-localization of TRPA1 expression (*red*) and isolectin B4 binding (*green*) is shown. Double-labeled neurons (*arrowheads*) are stained yellow in the merged panel. **b** Histogram shows the percent of TRPA1-positive neurons relative to isolectin B4-binding neurons. TRPA1 and isolectin B4 co-localization was significantly higher on day 2 after oxaliplatin administration (6 mg/kg, i.p.) compared to 5 % glucose treatment. Data are the mean ± SEM. n = 4 each for 5 % glucose and oxaliplatin administration (6 mg/kg, i.p.). *P < 0.05 versus 5 % glucose

We detected TRPA1 protein expression in DRG (L_4–6_) neurons at day 2 after oxaliplatin administration using immunohistochemistry. An increase in the frequency of TRPA1-positive cells was found in the oxaliplatin-treated rats compared with 5 % glucose-treated rats (Fig. 5a, b). Using computerized optical density image analysis, we measured the optical density of individual DRG neurons that were TRPA1 positive. Next, we compared isolectin B4-binding small neurons (green in Fig. 5a) between oxaliplatin- and 5 % glucose-treated rats. TRPA1 expression (red in Fig. 5a) overlapped with isolectin B4 binding to neurons. Immunofluorescence double-labeling experiments revealed a pronounced overlap between small-diameter DRG neurons expressing TRPA1 and isolectin B4 binding (yellow in Fig. 5a, merged). In 5 % glucose-treated rats, approximately half of the isolectin B4-binding DRG neurons were immunostained for TRPA1. The percent of TRPA1/isolectin B4 dual-positive cells relative to the total isolectin B4-binding neurons was significantly increased at day 2 after oxaliplatin administration (45.8 ± 5.0 vs. 64.7 ± 3.9 %, P < 0.05, n = 4) (Fig. 5b). Thus, oxaliplatin significantly increased expression of TRPA1 and isolectin B4 binding in DRG neurons.

### Oxaliplatin increases co-expression of TRPA1 mRNA and p-p38 in neurons

**Fig. 6:**
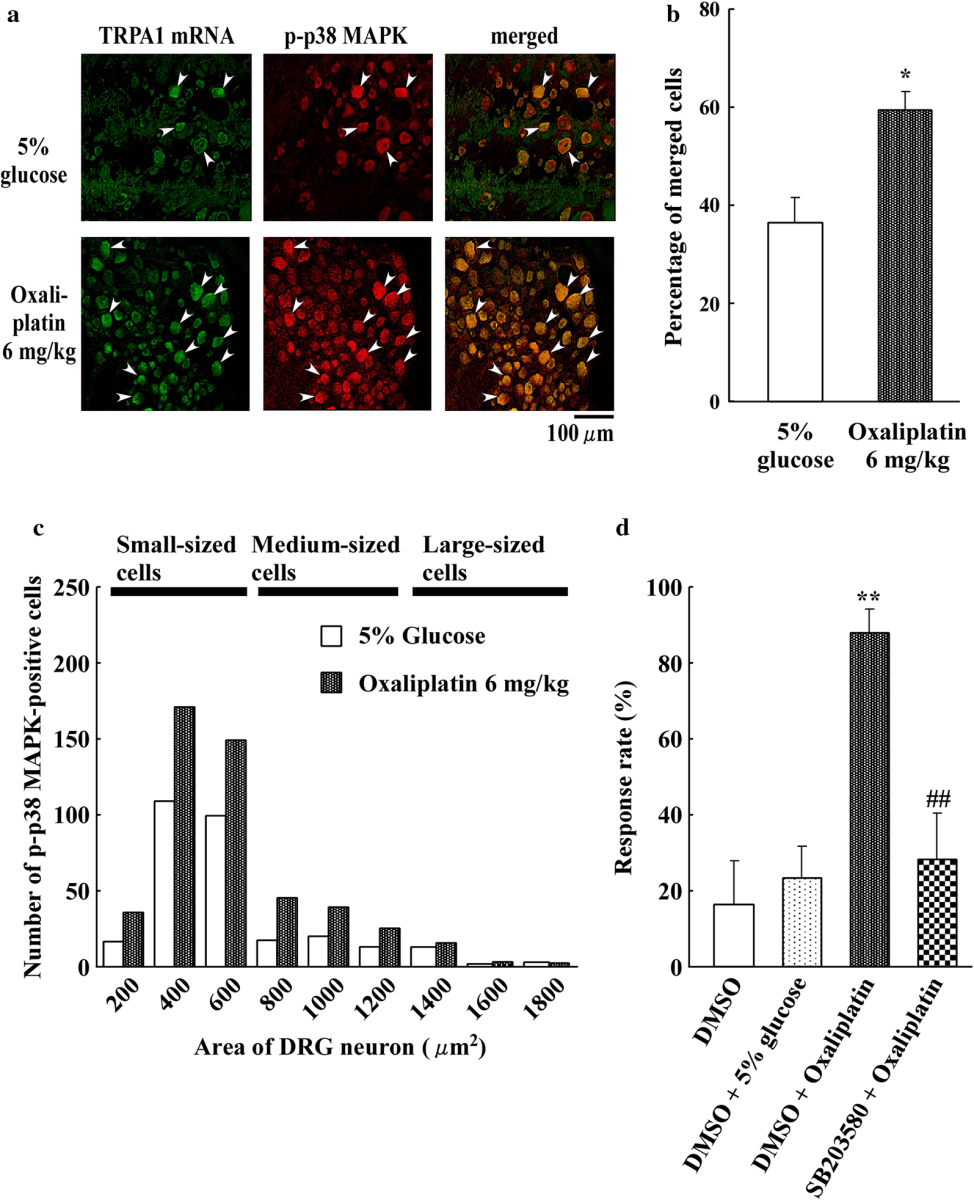
Effect of oxaliplatin on TRPA1 mRNA as seen with in situ hybridization histochemistry in rat DRG (L_4–6_) neurons. **a** Oxaliplatin (6 mg/kg, i.p.) increases the co-localization between TRPA1 and p-p38 MAP kinase in rat DRG (L_4–6_) neurons. The photomicrographs show representative data. Co-localization of TRPA1 mRNA (*green*) and p-p38 MAPK (*red*) is shown. Double-labeled neurons (*arrowheads*) appear yellow in the merged panel. **b** Histogram shows the percent of TRPA1 mRNA-positive neurons relative to p-p38-positive neurons. TRPA1 mRNA and p-p38 co-localization was significantly higher on day 2 after oxaliplatin administration (6 mg/kg, i.p.) compared to 5 % glucose treatment. Data are the mean ± SEM. n = 4 each for 5 % glucose and oxaliplatin administration. *P < 0.05 versus 5 % glucose. **c** Size distribution of TRPA1 mRNA- and p-p38 MAPK-co-expressing neurons in DRGs in 5 % glucose- and oxaliplatin-treated rats. TRPA1 mRNA and p-p38 MAPK-co-expressing neurons were distributed among all sizes of neurons, but most co-expression was found in small DRG neurons. Data are the mean ± SEM. n = 4 each for 5 % glucose treatment and oxaliplatin treatment (6 mg/kg, i.p.). **d** Intrathecal administration of the p38 MAPK inhibitor, SB203580 (0.5 μg μL^−1^ h^−1^), significantly prevents the acute cold hypersensitivity induced by oxaliplatin (6 mg/kg, i.p.). The acetone spray test at day 2 after oxaliplatin was measured in SB203580 and 20 % DMSO treatment groups. Data are the mean ± SEM. n = 4 each for 20 % DMSO treatment and oxaliplatin treatment (6 mg/kg, i.p.). **P < 0.01 versus DMSO. ^##^P < 0.01 versus oxaliplatin

We detected TRPA1 mRNA-labeled neurons in DRG (L_4–6_) neurons at day 2 after oxaliplatin administration using ISHH. We compared the expression of p-p38 (red in Fig. 6a) between oxaliplatin- and 5 % glucose-treated rats. TRPA1 mRNA-labeled neurons (green in Fig. 6a) overlapped with p-p38-positive neurons. Double-labeling experiments revealed a pronounced overlap between DRG neurons expressing TRPA1 mRNA and those expressing p-p38 (yellow in Fig. 6a, merged). The percent of TRPA1 mRNA/p-p38 dual-positive neurons relative to the total p-p38-positive neurons was significantly increased at day 2 after oxaliplatin administration (36.7 ± 5.0 vs. 59.6 ± 3.9 %, P < 0.01, n = 4) (Fig. 6b). Oxaliplatin significantly increased expression of TRPA1 mRNA and p-p38 in DRG neurons. TRPA1 mRNA/p-p38 dual-positive neurons in oxaliplatin-treated rats were mainly small-sized neurons compared to 5 % glucose treatment (Fig. 6c). Thus, oxaliplatin increased the number of small-diameter DRG neurons that co-expressed TRPA1 mRNA and p-p38.

### The effect of a p38 MAPK inhibitor (SB203580) on oxaliplatin-induced acute cold hypersensitivity

To examine the functional consequences of p38 MAPK activation, we investigated whether inhibition of p38 MAPK activation modifies the paw withdrawal response to oxaliplatin administration. To test this, rats were intrathecally treated with the MAPK inhibitor, SB203580 (0.5 μg μL^−1^ h^−1^), for 3 days before oxaliplatin (6 mg/kg) administration. The increase in oxaliplatin-induced acute cold hypersensitivity in the paw withdrawal response rate in the acetone spray test was significantly less in the SB203580 group at day 2 (Fig. 6d). As shown in Fig. 6d, 6 mg/kg oxaliplatin significantly increased the paw withdrawal response rate at day 2 compared to the DMSO control. We first showed that the p38 MAPK inhibitor (SB203580) reversed oxaliplatin-induced acute cold hypersensitivity.

## Discussion

Our data demonstrated that a single dose of oxaliplatin induced acute cold hypersensitivity in rats in a time- and dose-dependent manner. In previous behavioral studies, single or multiple doses of oxaliplatin produce cold allodynia/hyperalgesia [2, 4, 16]. The results of the present study agree with these previously published findings. In patients, cold allodynia induced by oxaliplatin is transient and usually improves within 3–4 days [22]. However, in animals, the response to the acetone spray test or cold plate test is significantly increased with 6 mg/kg oxaliplatin from day 1 through day 7 after a single dose of oxaliplatin [4, 17, 23, 24]. Thus, one limitation of our study is this difference between the animal model and patients. However, the discrepancy between humans and rodents may be explained by differences in the dose, period of administration, and testing methods.

In the present study, we showed that a single dose of oxaliplatin increased TRPA1 mRNA and protein in rat DRGs. TRPA1 mRNA was observed 1 and 2 days after injection and then gradually decreased, whereas TRPA1 protein increased 2 days after injection. Expression peaked on day 4 and was maintained until day 7 after injection. A recent study reported a transient increase in TRPA1 mRNA only 6 h after exposure of cultured rat DRG neurons to oxaliplatin [25]. Moreover, TRPA1 mRNA is moderately and significantly increased in mouse DRGs after a single dose of oxaliplatin (3 mg/kg), but no further increase is observed after 6 h [16]. TRPA1 mRNA levels, but not TRPM8 mRNA, are slightly increased in mouse DRGs 90 h after oxaliplatin (6 mg/kg) administration [26]. Moreover, TRPA1 mRNA expression levels are significantly increased in rat DRGs on day 3 after oxaliplatin (6 mg/kg) treatment [27]. Our data in this study revealed that TRPA1 mRNA remained elevated from day 1 to 4 after a single dose of oxaliplatin (6 mg/kg), whereas TRPA1 protein was observed from day 2 to 7. The time course of acute cold hypersensitivity (peak effect was observed at day 2) may be explained by the increase in TRPA1 mRNA and protein in DRGs. Thus, TRPA1 appears to be involved in acute cold hyperalgesia induced by oxaliplatin. In addition, using ISHH, we found that oxaliplatin increased TRPA1 mRNA expression in rat DRG small neurons after days 1, 2, and 4. TRPA1 mRNA-positive DRG neurons were mainly small-sized neurons. Taken together, our results support the suggestion that molecular biological data for TRPA1 are related to the behavioral cold test. Thus, the sustained cold hypersensitivity induced by oxaliplatin, as observed by the ratio of neurons expressing TRPA1, increased after day 2. TRPA1 up-regulation likely plays an important role in nociceptive processing in oxaliplatin-induced peripheral acute cold hypersensitivity.

To determine whether small neurons expressed TRPA1 protein, DRG sections were double-labeled for TRPA1 and isolectin B4. The ratio of neurons expressing TRPA1 among those that were isolectin B4 positive was significantly higher in oxaliplatin-treated rats, confirming that a considerable number of C-fiber neurons began to express TRPA1 after oxaliplatin administration. TRPA1 expression is seen in many small myelinated axons (Aδ fibers, 16.1 % of TRPA1 axons) as well as in unmyelinated axons (C fibers, 78 %) [27]. Thus, oxaliplatin increased the expression of TRPA1 in small DRG neurons, and TRPA1 was responsible for oxaliplatin-induced acute cold hypersensitivity. Kim and colleagues showed that almost half of the TRPA1-positive neurons in the trigeminal ganglion bind IB4 [27]. Moreover, Joseph et al. showed that intrathecal administration of the neurotoxin IB4-saporin, which selectively blocks IB4-positive nociceptors, completely prevented oxaliplatin-induced cold allodynia/hyperalgesia [28]. Taken together, our data suggest that oxaliplatin may be involved in the increase in TRPA1 in DRG small neurons, resulting in acute cold hypersensitivity. Furthermore, cold hypersensitivity induced by a single dose of oxaliplatin is inhibited in *Trp1*^−*/*−^ mice [16]. Previous behavioral studies have shown that intrathecal administration of antisense TRPA1 inhibits inflammation and prevents nerve injury-induced cold hyperalgesia [19]. Consistent with these reports, we confirmed that oxaliplatin-induced acute cold hypersensitivity in rats on days 1, 2, and 4 was significantly inhibited by intrathecal administration of TRPA1 AS-ODN.

Several reports have demonstrated that the activation of p38 MAPK in DRG neurons is induced by not only peripheral inflammation but also axotomy, and corresponding nociceptive behaviors are prevented when p38 MAPK is inhibited [20, 29, 30]. Moreover, the effects of a p38 MAPK inhibitor on cold hyperalgesia are mediated by inhibition of p38 MAPK activation in the DRG [19]. The neurotoxicity of platinum derivatives is strictly linked to modifications induced by oxaliplatin on activation of the MAPK pathway [31]. In addition, noxious cold stimulation induces stimulus intensity-dependent p38 MAPK activation predominantly in TRPA1-expressing DRG neurons. For example, the activation of p38 MAPK pathways in DRG neurons by gastric distension-induced visceral pain may be correlated with the activation state of primary afferent neurons through TRPA1 [31]. In vitro, administration of oxaliplatin induces dose-dependent activation by phosphorylation of p38 MAPK in DRG neurons [32]. In the present study, we found that the expression of p-p38 MAPK was co-localized with TRPA1 expression in small DRG neurons after oxaliplatin treatment. Intrathecal administration of the p38 MAPK inhibitor, SB203580, prevented the oxaliplatin-induced acute cold hypersensitivity. Taken together, these findings suggest that the activation of p38 MAPK pathways in the DRG by administration of oxaliplatin may be, at least in part, correlated with functional activity through TRPA1, and further, involved in the development of thermal hyperalgesia.

## Conclusion

The present data suggest that up-regulation of TRPA1 in small neurons of DRGs probably plays a crucial role in the pathogenesis of acute cold hypersensitivity via activation of p38 MAPK after oxaliplatin administration. Our data also suggest that activation of p38 MAPK in the DRG may be a main mechanism of up-regulation of TRPA1 in small DRG neurons during the process of oxaliplatin-induced acute cold hypersensitivity.

## Methods

### Experimental animals

Male Wistar rats weighing 250–330 g (Japan Laboratory Animals, Inc., Tokyo, Japan) were used. All rats were housed individually under automatically controlled environmental conditions, using a 12-h light–dark cycle (lights on from 08:00 to 20:00) with free access to food and water. All animals were quarantined in centralized animal facilities for at least 7 days upon arrival. Each animal was used only once. Experiments were carried out according to the guidelines for animal care and use published by the National Institutes of Health and the committee of Showa Pharmaceutical University.

### Drug administration

One dose (1, 3, or 6 mg/kg) of oxaliplatin (Elplat^R^) was intraperitoneally (i.p.) administered (Yakult Co., Ltd., Tokyo, Japan). Oxaliplatin was dissolved in a 5 % glucose solution at a concentration of 2 mg/ml depending on the animal’s weight, to ensure intraperitoneal injections of less than 2.5 ml. The control groups were injected with 5 % glucose solution. Volumes of the 5 % glucose solution were adjusted to the weight of each rat and injected by the same route in the control group.

### Acetone spray test for acute cold hypersensitivity

Observers blinded to the experimental conditions tested the rats using the acetone/water spray tests at the same time on days 0–7 after oxaliplatin treatment. To estimate cold sensitivity of the paw, acetone (Kanto Chemical Co., Inc., Tokyo, Japan) or water (22 °C) was used, according to modification of previously described methods [33]. Rats were placed in a clear plastic box (23 × 23 × 12 cm) with a wire mesh floor and allowed to habituate for 30 min prior to testing. After habituation, 50 μl fluid (acetone or water) was sprayed on the plantar surface of the hind-paws five times using a MicroSprayer^®^ (Penn Century Inc., Philadelphia, PA, USA). Paw withdrawal, defined as flinching, licking, or biting of the limb, was measured for 1 min after the start of the acetone spray. For the water spray test, mineral water at 22 °C was used. The effects of acetone or water spray were repeatedly evaluated over time after oxaliplatin (1, 3, and 6 mg/kg) or 5 % glucose administration. A group of animals given oxaliplatin or glucose was tested 1 day before drug administration and for 7 days after drug administration.

### Quantitative real-time polymerase chain reaction (RT-PCR)

Rats were deeply anesthetized with pentobarbital (50 mg/kg, i.p.) on days 1, 2, 4, or 7 after oxaliplatin administration (6 mg/kg, i.p.), and DRGs (L_4–6_) were removed. RNA was purified using TRIzol (Invitrogen, Carlsbad, CA, USA). Total RNA (1 μg) was used for cDNA synthesis with a SuperScript^®^ VILO™ cDNA Synthesis Kit (Invitrogen). Quantitative RT-PCR was performed using an Applied Biosystems StepOne™ RealTime PCR System (Applied Biosystems, Tokyo, Japan), using EXPRESS SYBR^®^ GreenER™ qPCR SuperMixes^®^ and Two-Step qRT-PCR kits (Invitrogen), according to the manufacturer’s instructions. The cycling conditions for all primers were as follows: 2 min at 50 °C to incubate uracil DNA glycosylase, 2 min at 95 °C, followed by 50 cycles consisting of two steps: 15 s at 95 °C (denaturation) and 1 min at 60 °C (annealing and extension). TRPA1 levels were evaluated by comparison with β-actin levels. Primer sequences were as follows: TRPA1, (forward) 5′-GGCATGTACAACGAAGTGATCAA-3′ and (reverse) 5′-CTGTGTTCCCATTCTCTCCTTCTAAA-3′ corresponding to the rat TRPA1 gene (GenBank: AY496961.1) and β-actin, (forward) 5′-CAGGTCATCACTATCGGCAATG-3′ and (reverse) 5′-GAGACTACAACTTACCCAGGAAGGAA-3′ corresponding to the rat β-actin gene (Sigma-Aldrich, Inc., St. Louis, MO, USA). In all cases, the validity of amplification was confirmed by the presence of a single peak in the melting temperature analysis and linear amplification during the PCR cycles.

### Western blot analysis

On days 2, 4, and 7 after oxaliplatin (6 mg/kg, i.p.) administration, rats were deeply anesthetized with pentobarbital (50 mg/kg, i.p.). DRGs (L_4–6_) were collected and homogenized in cold extraction buffer consisting of 10 mM Tris–HCl buffer at pH 7.5, 100 mM NaCl, 1 mM EDTA, 0.5 % Triton X-100, and 0.5 % deoxycholate. The homogenates were centrifuged for 30 min at 15,000×*g* at 4 °C, and the supernatant was collected. Total protein of the supernatant (30 μg) was electrophoresed on an SDS–polyacrylamide gel (7.5 %), and separated proteins were transferred onto polyvinylidene fluoride membranes. Anti-TRPA1 antibody (Alomone Labs, Jerusalem, Israel) diluted 1:200 was used, and anti-β-actin antibody (Sigma-Aldrich) was used as an internal control. Horseradish peroxidase-labeled anti-rabbit antibody diluted 1:2000 was used as the secondary antibody (Sigma-Aldrich). Specific bands were detected using enhanced chemiluminescence plus the TM Western Blotting Detection Kit (GE Healthcare, Buckinghamshire, UK) according to the manufacturer’s protocol. The intensities of immunoreactive bands were analyzed with MultiGage Ver.3 software (Fuji Film, Tokyo, Japan).

### Immunohistochemistry

Rats were deeply anesthetized with pentobarbital (50 mg/kg, i.p.) on day 2 after oxaliplatin administration (6 mg/kg, i.p.). Rats were perfused transcardially with 20 ml potassium-free phosphate-buffered saline (K^+^-free PBS; pH 7.4) followed by 50 ml 4 % paraformaldehyde solution. The DRGs (L_4–6_) were removed, post-fixed for 3 h, cryoprotected overnight in 25 % sucrose solution, and stored at −80 °C until use. DRGs were cut at 10 μm thickness, thaw-mounted on silane-coated glass slides, and air-dried overnight at room temperature. DRG sections were incubated with excess blocking buffer containing 2 % skim milk in 0.1 % Triton X-100 in K^+^-free PBS and subsequently reacted overnight at 4 °C with anti-TRPA1 antibodies (Alomone Labs, 1:200) in 2 % bovine serum albumin/0.1 % Triton X-100 in K^+^-free PBS. The sections were then incubated in fluorescein isothiocyanate-conjugated anti-rabbit IgG (Sigma-Aldrich, 1:100) for 1 h at room temperature. Double labeling studies for TRPA1 and isolectin B4 from *Bandeiraea simplicifolia* were performed using an immunofluorescent procedure. Sections were incubated with anti-TRPA1 antibody and FITC-conjugated isolectin B4 (Enzo Life Science, Ontario, Canada) in blocking buffer for 4 h at 4 °C. After washing with PBS, sections were incubated with Cy3-conjugated anti-goat antibody (Bethyl Laboratories, Inc., Montgomery, TX, USA).

All sections were treated with Permafluor (Thermo Shandon, Pittsburgh, PA, USA) and cover-slipped and evaluated with an Olympus laser-scanning confocal microscope (FLUOVIEW BW50, Olympus, Tokyo, Japan) at wavelengths of 488 nm and 568 nm. A total of five sections (90 μm apart) were randomly selected from each DRG. The proportion of TRPA1-positive cells was calculated according to the size of the cell body. At least 350 neurons from each DRG (L_4–6_) of each rat were measured.

### In situ hybridization histochemistry (ISHH)

On day 2 after oxaliplatin (6 mg/kg, i.p.), rats were deeply anesthetized with pentobarbital (50 mg/kg, i.p.), perfused with 4 % paraformaldehyde, and lumbar DRGs (L_4–6_) were rapidly dissected. Following post-fixation and cryo-protection in 30 % sucrose in PBS, single DRGs were embedded in OCT, frozen at −80 °C, and sectioned at 10 μm thickness. Sections were thaw-mounted onto MAS-coated glass slides (Matsunami Glass Inc., Ltd., Osaka, Japan) and fixed in 4 % paraformaldehyde in PBS for 10 min. After washing in PBS, the sections were treated with 1 mg/ml proteinase K (Sigma-Aldrich) in PBS for 10 min at room temperature, post-fixed in the same fixative, acetylated with acetic anhydride in 0.1 M triethanolamine, prehybridized for 60 min at 55 °C, and hybridized with digoxygenin (DIG)-labeled RNA probes overnight at 55 °C. DIG-labeled sense and anti-sense RNA probes corresponded to nucleotides 302–788 of rat TRPA1 mRNA (AY496961). Following post-hybridization washes and blocking, sections were incubated for 120 min in anti-DIG antibody conjugated to alkaline phosphatase (1:5000; Roche, Mannheim, Germany), and signal was visualized using nitro blue tetrazolium/bromochloroindolyl phosphate substrates (Roche). An equivalent sense probe displayed no signal.

### Fluorescence in situ hybridization (FISH) and immunofluorescence double staining

For double staining using FISH and immunofluorescence, the OCT-embedded DRG sections described above were first incubated with DIG-labeled sense and anti-sense RNA probes corresponding to nucleotides 302–788 of rat TRPA1 mRNA (AY496961), and then incubated with anti-phospho-p38 (p-p38) MAPK (Thr180/Tyr182)(3D7) rabbit mAb (1:50; Cell Signaling Technology, Danvers, MA, USA). Sections were incubated with anti-DIG-FITC to detect TRPA1 mRNA and 2 µg/ml Alexa Fluor^®^ 594-labeled secondary antibody (Molecular Probes, Ina, Eugene, OR, USA) to detect phospho-p38 MAPK.

### Image analysis

Signals were analyzed with fluorescence microscopy at 400× magnification using a microscopy-digital camera system. Experimenters who were unaware of the experimental protocol counted cells in a blinded manner. A total of five sections (90 μm apart) were randomly selected from each DRG. The ratio of TRPA1-positive cells in the total profile was calculated for days 0, 1, 2, 4, and 7 after oxaliplatin or 5 % glucose treatment. Signal intensity and area frequency analysis of each neuron were calculated with ImageJ 1.46. Neurons were considered TRPA1 positive if their signal intensity was threefold higher than background. For the background, we measured the signal intensity in three areas outside the DRG and calculated the mean signal intensity. The proportion of TRPA1-positive cells of the total was calculated according to the size of the cell body. At least 900 neurons from each DRG (L_4–6_) of each rat were measured.

### Intrathecal injection of AS-ODN or the p38 MAPK inhibitor

Under sodium pentobarbital (50 mg/kg) anesthesia, the rat atlanto-occipital membrane was cut. A soft tube (Silascon, Kaneka Medix Company, Osaka, Japan; outer diameter, 0.64 mm) was inserted into the subarachnoid space for a length of 8.0 cm to ensure that the tip reached the lumbar enlargement. The end part of the catheter was inserted caudally into the subarachnoid space through a small slit in the atlanto-occipital membrane to extend 8.0 cm beyond the slit. The rostral part of the catheter was sutured to the occipital muscle to immobilize the catheter, and the wound was closed in two layers with 3-0 silk thread. To obtain sustained infusion of AS-ODN targeting TRPA1 (0.5 nmol μL^−1^ h^−1^) or MM-ODN (0.5 nmol μL^−1^ h^−1^), an ALZET^®^ osmotic pump (7-day pump, 1 μL/h; DURECT) was filled with AS-ODN (5′-TCTATGCGGTTATGTTGG-3′) or MM-ODN (5′-ACTACTACACTAGACTAC-3′) in saline. ODNs were intrathecally infused for 3 days, and then rats were given oxaliplatin or 5 % glucose. Rats that showed motor impairment were excluded from further analysis. Whether ODNs can reach DRGs in sufficient concentrations by intrathecal delivery has been frequently questioned. However, several reports have demonstrated that intrathecally delivered ODNs accumulate in DRG cells [34, 35].

An ALZET osmotic pump (7-day pump, 1 μL/h; DURECT) filled with the p38 inhibitor, SB203580 (0.5 μg μL^−1^ h^−1^) (A.G. Scientific, Inc., San Diego, CA, USA), in 20 % dimethylsulfoxide (DMSO) was connected to the soft tube. SB203580 was intrathecally infused for 3 days, and then rats were given oxaliplatin or 5 % glucose. Rats that showed motor impairment were excluded from further analysis.

### Statistical analysis

All data are expressed as the mean ± SEM. For the time course study of acute cold hypersensitivity after oxaliplatin administration, the significance of the difference among the groups was analyzed by two-way ANOVA, followed by Dunnett’s multiple comparison test. For other multiple group analyses, such as the effect of oxaliplatin on TRPA1 mRNA and protein expression, the significance of the difference among the groups was evaluated with one-way ANOVA, followed by Dunnett’s multiple comparison test. For analysis of two groups, such as vehicle versus oxaliplatin or MM-ODN versus AS-ODN, the significance of the difference between the groups was determined using the F-test, followed by Student’s or Aspin-Welch’s *t* test. Statistical significance was established at P < 0.05.

## Abbreviations

ANOVA: analysis of variance
AS-ODN: antisense oligodeoxynucleotides
DIG: digoxygenin
DMSO: dimethylsulfoxide
DRG: dorsal root ganglion
FISH: fluorescence in situ hybridization
MAPK: p-p38 mitogen-activated protein kinase
MM-ODN: mismatched oligodeoxynucleotides
RT-PCR: quantitative real-time polymerase chain reaction
TRPA1: transient receptor potential ankyrin 1
